# Electro-rhinosculpture for the surgical management of rhinophyma

**DOI:** 10.1308/rcsann.2014.96.1.81

**Published:** 2014-01

**Authors:** GAC Wheble, F Ahmed, AN Pandya

**Affiliations:** Portsmouth Hospitals NHS Trust,UK

**None of the authors have any commercial associations or financial disclosures that might pose or create a conflict of interest.**

We describe a simple technique for excision of rhinophyma using equipment found commonly in theatre. The nose is infiltrated with lignocaine and adrenaline, and a colposcopic loop electrode (15mm disposable loop electrode; Meditech, Shaftesbury, UK) is used to shave excess tissue in smooth planes while providing simultaneous haemostasis. The burr (6mm Bud Trimmer Burr; Mercian, Bromsgrove, UK) is then used to soften the nasal contours into the desired appearance. Additional haemostasis is performed with loop diathermy before the wound is dressed.

Excision of rhinophyma is typically a bloody and time consuming affair. Our technique creates a smoothly contoured appearance and simultaneous haemostasis with only a few passes of the loop cautery tip.

**Figure 1:**
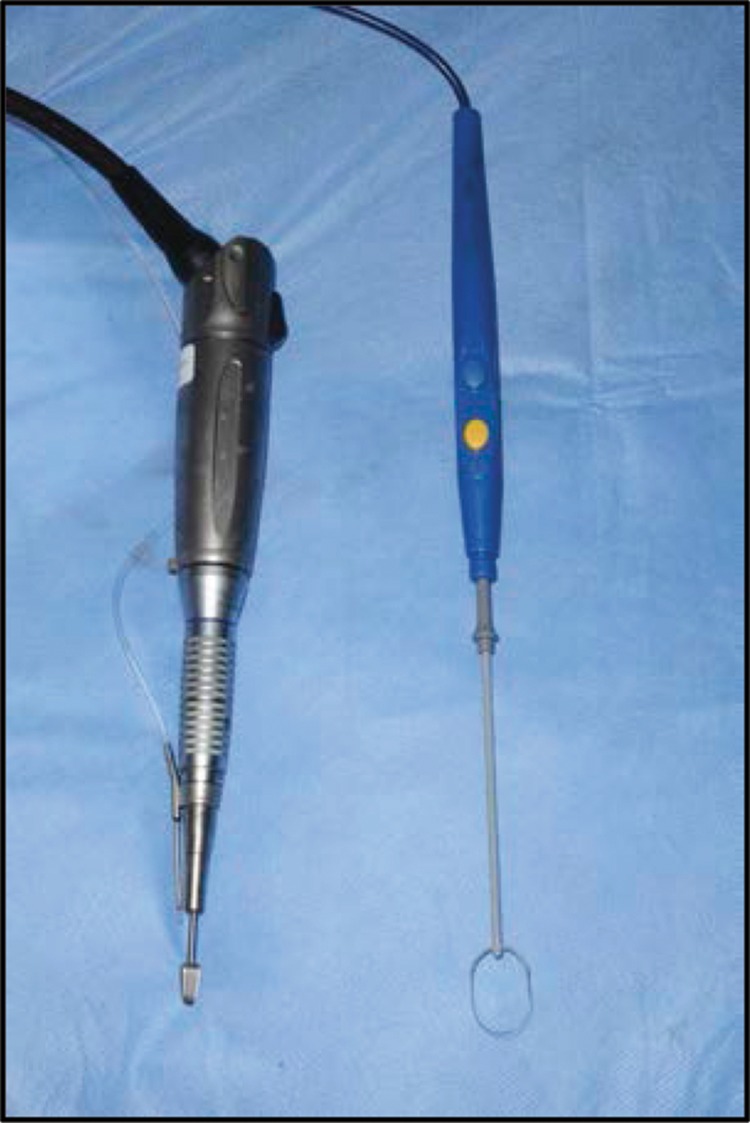
Disposable loop electrode. (Meditech, 15mm) and Bud Trimmer Burr (Mercian Surgical Ltd, 6.0mm with Aesculap Drill System)

**Figure 2:**
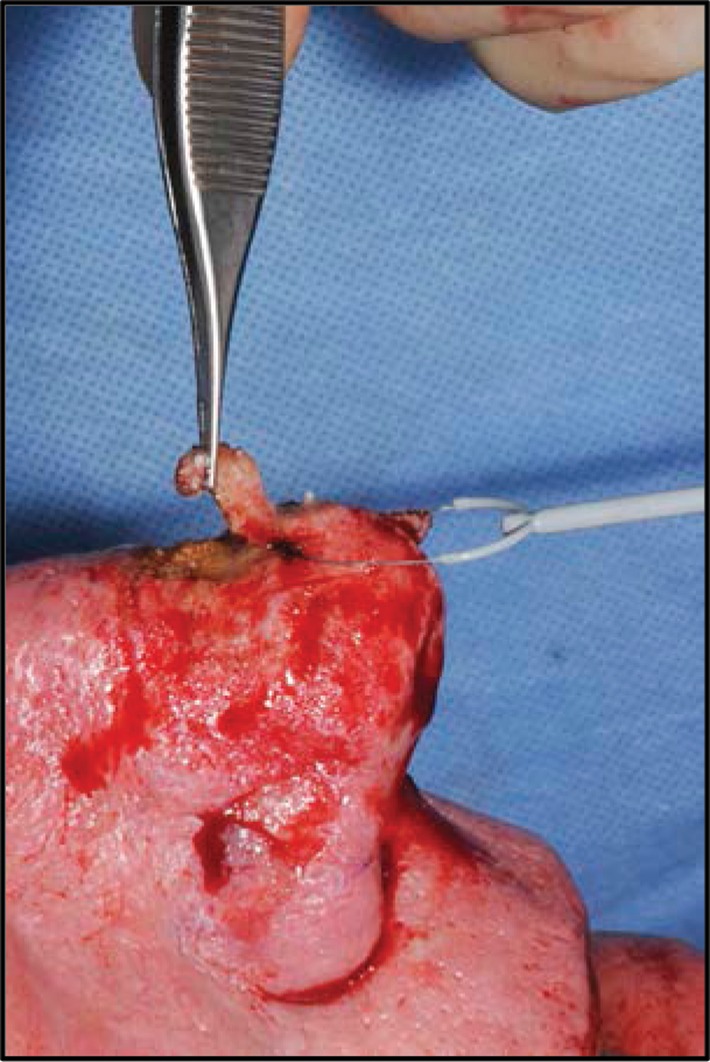
Demonstration of use of colposcopic loop cautery

**Figure 3:**
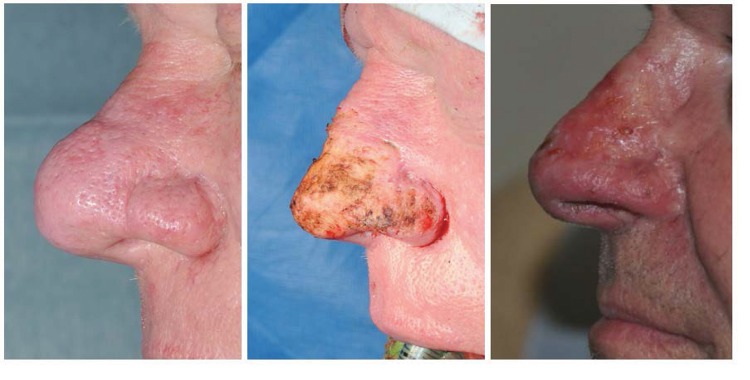
Pre-op (left), immediately post-op (centre) and 6 weeks post-op (right) lateral views

